# Clock-controlled mir-142-3p can target its activator, *Bmal1*

**DOI:** 10.1186/1471-2199-13-27

**Published:** 2012-09-07

**Authors:** Xiaochao Tan, Peng Zhang, Lan Zhou, Bin Yin, Hui Pan, Xiaozhong Peng

**Affiliations:** 1 State Key Laboratory of Medical Molecular Biology, Institute of Basic Medical Sciences, Chinese Academy of Medical Sciences and Peking Union Medical College, Beijing, 100005, China; 2 Department of Physiology, Institute of Basic Medical Sciences, Chinese Academy of Medical Sciences and Peking Union Medical College, Beijing, 100005, China; 3 Department of Endocrinology, Peking Union Medical Hospital, Chinese Academy of Medical Sciences and Peking Union Medical College, Beijing, 100730, China

**Keywords:** mir-142-3p, Bmal1, Circadian clock

## Abstract

**Background:**

microRNAs (miRNAs) are shown to be involved in the regulation of circadian clock. However, it remains largely unknown whether miRNAs can regulate the core clock genes (*Clock* and *Bmal1*).

**Results:**

In this study, we found that mir-142-3p directly targeted the 3’UTR of human *BMAL1* and mouse *Bmal1*. The over-expression (in 293ET and NIH3T3 cells) and knockdown (in U87MG cells) of mir-142-3p reduced and up-regulated the *Bmal1/BMAL1* mRNA and protein levels, respectively. Moreover, the expression level of mir-142-3p oscillated in serum-shocked NIH3T3 cells and the results of ChIP and luciferase reporter assays suggested that the expression of mir-142-3p was directly controlled by CLOCK/BMAL1 heterodimers in NIH3T3 cells.

**Conclusions:**

Our study demonstrates that mir-142-3p can directly target the 3’UTR of Bmal1. In addition, the expression of mir-142-3p is controlled by CLOCK/BMAL1 heterodimers, suggesting a potential negative feedback loop consisting of the miRNAs and the core clock genes. These findings open new perspective for studying the molecular mechanism of circadian clock.

## Background

Daily patterns of physiological and behavioral processes can be observed in almost all organisms, ranging from cyan bacterium to humans. The circadian clock plays important roles in local and systemic physiology and in pathology [1-3]. The molecular machinery of the circadian clock is thought to be composed of self-sustaining transcriptional feedback loops. The core proteins of the molecular pathway regulating the circadian clock are CLOCK and BMAL1 [4,5], which form heterodimers that drive the transcription of circadian output genes (CCGs) [6,7]. The disruption of the expression of these two core molecules leads to remarkable changes in the circadian clock. The mutation of the *Clock* gene (*Clock*Δ19/19) or the deletion of the *Bmal1* gene (*Bmal1*−/−) abolishes circadian oscillations [4,5]. However, *Clock* knock-out mice exhibit a normal physiological rhythm because of compensation by NPAS2 [8,9]. CCGs, such as *Per* and *Cry*, can weaken the transcriptional activity of CLOCK/BMAL1 heterodimers, forming a negative feedback loop called the core loop [10]. *Rev-Erbα* and *RORα* bind to an ROR element in the promoter of *Bmal1*, inhibiting or activating its transcription, respectively, which is called the stabilizing loop [11].

MicroRNAs (miRNAs) are small non-coding RNA molecules that regulate the expression of target genes at the post-transcriptional level. The roles of miRNAs in the circadian clock have been discussed in several reports. Xu et al. showed that the levels of specific miRNAs (e.g., mir-96, mir-124a, mir-103, mir-182 and mir-106b) oscillate in the mouse retina in a circadian pattern [12]. Clock-controlled mir-219 acts in the fine tuning of the length of the circadian period, and light-induced mir-132 was shown to be a negative regulator of the light-dependent resetting of the clock [13]. The mir-192/194 cluster was identified as a potent inhibitor of the *Per* gene family, the enforced expression of which leads to an altered circadian rhythm [14]. Kadener et al. found that a miRNA, the developmental regulator *bantam*, plays a role in the core circadian pacemaker by targeting *clock* in *Drosophila*[15]. In addition, mir-206 was shown to be a regulator of the circadian clock in skeletal muscle [16]. Recently, a report showed that mir-494 and mir-142-3p, two circulating miRNAs, can target the *Bmal1* 3’ UTR in mice [17]. Thus, mounting evidence suggests that miRNAs act as very important regulators of the circadian clock [18].

We are interested in the miRNAs which can directly target the core clock regulators, *Clock* and *Bmal1*, and prediction, followed by experimental validation, was performed to identify potential *Clock/Bmal1*-targeting miRNAs. The level of CLOCK protein remains nearly unchanged during the circadian cycle [19], and it can be functionally compensated by NPAS2. Therefore, we focused on the potential *Bmal1*-targeting miRNAs. Here in this study, we found that mir-142-3p directly targeted *Bmal1* and its expression was regulated by CLOCK/BMAL1 heterodimers.

## Results

### Mir-142-3p is a *Bmal1*-targeting miRNA

**Figure 1:**
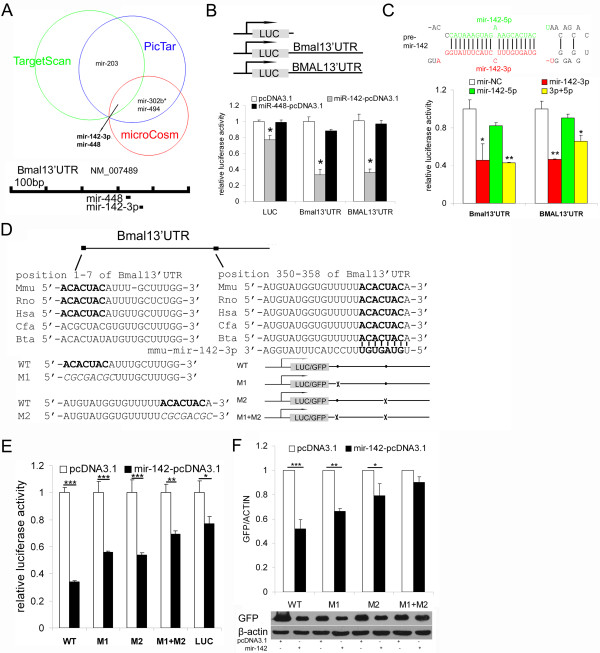
**mir-142-3p can target *Bmal1 *3’UTR. **(**A**) Prediction results using three bioinformatic algorithms (TargetScan, PicTar and MicroCosm). The schematic representation of the binding sites for mir-142-3p and mir-448 in the 3’ UTR of *Bmal1 *is shown on the bottom. (**B**) A luciferase reporter assay was employed to screen the miRNAs that could target the mouse *Bmal1 *and human *BMAL1 *3’ UTR. 293ET cells were co-transfected with luciferase reporter plasmids and mir-142 or mir-448 expression plasmids or control vector, and the normalized firefly luciferase activity was measured (mean ± SD, n = 3). (**C**) A luciferase reporter assay was employed to confirm that mir-142-3p but not mir-142-5p can target *Bmal1 *in 293ET cells. Synthetic mir-142-3p mimics or mir-142-5p mimics or control microRNA mimics (mir-NC) were co-transfected with luciferase reporter plasmids into 293ET cells and the normalized firefly luciferase activity was measured (mean ± SD, n = 4). The top sketch modified from the miRBase shows that the pre-mir-142 can produce two mature miRNAs, mir-142-3p and -5p. (**D**) Schematic representation of the mir-142-3p binding sites in the *Bmal1 *3’ UTR and mutation of the mir-142-3p binding sites. In addition to the conserved binding site at position 350–358 of the *Bmal1 *3’ UTR, there is a poorly conserved binding site at position 1–7. (**E**) 293ET cells were transfected with the empty luciferase reporter vector (LUC) or luciferase reporter plasmids containing the *Bmal1 *3’ UTR with no mutations (WT) or with mutations (M1,M2 and M1 + M2) in the mir-142-3p binding sites and normalized luciferase activity was measured (mean ± SD, n = 4). (**F**) GFP reporter constructs containing the wild-type *Bmal1 *3’ UTR or mutated sequences were co-transfected into 293ET cells with mir-142-pcDNA3.1 or empty vector (pcDNA3.1), and the expression of GFP was determined by western blotting. The western blotting results were quantified from the pixel values in grayscales (mean ± SD, n = 3). Representative western blotting results are shown (bottom). *P < 0.05, **P < 0.01 and ***P < 0.001.

By computational prediction using three algorithmic methods (TargetScan, PicTar and MicroCosm), we found two miRNAs that putatively target *Bmal1* (Figure 1A). These two miRNAs (mir-142-3p and mir-448) were subjected to validation by luciferase reporter assays using reporters containing the 3’UTR of mouse *Bmal1 *or human *BMAL1*. The mir-142-pcDNA3.1 and mir-448-pcDNA3.1 expression plasmids were first transfected into 293ET cells and the expressions of mature miRNAs were verified by quantitative RT-PCR (Additional file 1A). Over-expression of mir-142 but not mir-448 significantly repressed the activity of the *Bmal1*/*BMAL1* 3’UTR-luciferase reporter (Figure 1B). We also found that over-expression of mir-142 could significantly reduce the luciferase reporter RNA levels (Additional file 1B), suggesting that mir-142 was able to accelerate target mRNA degradation. Because pre-mir-142 can produce two mature miRNAs (mir-142-3p and mir-142-5p) (Figure 1C, top), a luciferase reporter assay was performed to ensure that mir-142-3p was the relevant effector. Indeed, the results confirmed that mir-142-3p but not mir-142-5p repressed the *Bmal1*/*BMAL1* 3’UTR-luciferase reporter activity (Figure 1C, bottom).

As there are two mir-142-3p binding sites in the 3’ UTR of *Bmal1*, we mutated one or both of them to determine which site was functional, even though the first binding site (position1-7) is poorly conserved among mammals (Figure 1D). In luciferase reporter assays, mutating each of the two mir-142-3p binding sites significantly reduced the repression effect of mir-142 over-expression on luciferase activity, and double mutations (M1 + M2) showed greater effect than single mutation (M1 or M2) (Figure 1E). The luciferase activity of (M1 + M2) was still decreased by about 30% with mir-142 over-expression, which may be ascribed to the background of luciferase reporter experiments for that mir-142 over-expression also led to an approximately 25% reduction of luciferase activity of the empty reporter vector (LUC) (Figure 1E). In GFP reporter assays, we got very similar results that each of the binding site was functional for the interaction between mir-142-3p and *Bmal1*3’UTR (Figure 1F).

### Mir-142-3p regulates the *Bmal1* mRNA and protein levels

**Figure 2:**
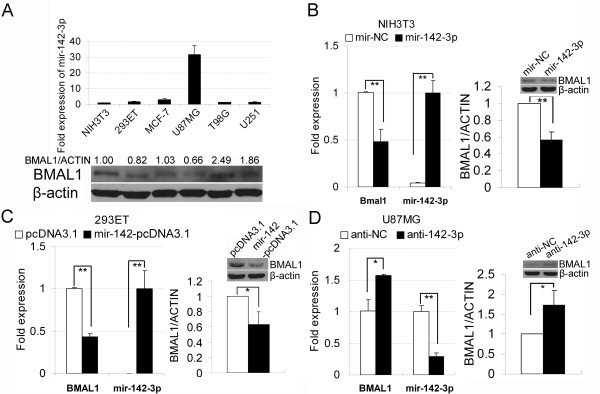
**mir-142-3p regulates the *Bmal1 *mRNA and protein levels. **(**A**) Total cell RNA and protein were extracted from the six cell lines (NIH3T3, 293ET, MCF-7, U87MG, T98G and U251), and the expression of mir-142-3p as well as the BMAL1 protein were determined by quantitative RT-PCR (mean ± SD, n = 3) and western blotting, respectively. (**B**-**C**) Ectopic expression of mir-142-3p in NIH3T3 and 293ET cells inhibited the expression of *Bmal1*/*BMAL1*. Due to the low transfection efficiency of plasmids, the NIH3T3 cells were transfected with synthetic mir-142-3p mimics (mir-142-3p) or control mimics (mir-NC) (**B**). The 293ET cells were transfected with the mir-142 expression plasmid (mir-142-pcDNA3.1) or control vector (pcDNA3.1) (**C**). The over-expression of mir-142-3p in NIH3T3 and 293ET cells was first verified, and then the *Bmal1*/*BMAL1 *mRNA and protein levels were detected by quantitative RT-PCR (mean ± SD, n = 3) and western blotting, respectively. The expression levels of mir-142-3p in control NIH3T3 (mir-NC) and 293ET (pcDNA3.1) cells were normalized against that in mir-142-3p/mir-142-pcDNA3.1-transfected cells which were set at 1. Note that, because the mir142-3p expression was low in control 293ET cells (pcDNA3.1) and was increased more than 1000-fold when cells were transfected with mir-142-pcDNA3.1, the relative expression level of mir-142-3p in control group (pcDNA3.1) was very low. (**D**) The endogenous mir-142-3p in U87MG cells was knocked down by transfection with synthetic mir-142-3p antagomirs (anti-142-3p). The expression level of mir-142-3p and *BMAL1 *mRNA in U87MG cells were determined by quantitative RT-PCR (mean ± SD, n = 3) and normalized against controls set at 1. All of the western blotting results were quantified from the pixel values in grayscales (mean ± SD, n = 3) and normalized against controls set at 1. Inserts are the representative western blotting results. *P < 0.05 and **P < 0.01.

To investigate the regulatory effect of mir-142-3p on the expression of endogenous Bmal1, we detected the expression level of mir-142-3p and Bmal1 protein level in six cell lines (NIH3T3, 293ET, MCF-7, U87MG, T98G and U251) generally used in our laboratory. We found that the level of mir-142-3p was extremely high and the protein level of Bmal1 was low in U87MG cells compared with the other five cell lines. In contrast, NIH3T3, 293ET, MCF-7, T98G and U251 cells with low mir-142-3p levels expressed high levels of Bmal1 protein (Figure 2A). To confirm that mir-142-3p can regulate the expression of *Bmal1*, mir-142-3p was over-expressed in the NIH3T3 and 293ET cells or knocked down in U87MG cells, and the mRNA and protein levels of human *BMAL1* and mouse *Bmal1* were determined. The over-expression of mir-142-3p led to reductions in the levels of both *Bmal1* (*BMAL1*) mRNA and protein in NIH3T3 (Figure 2B) and 293ET cells (Figure 2C), and knocking down mir-142-3p with antagomirs increased the expression of BMAL1 mRNA and protein in U87MG cells (Figure 2D). These results showed that mir-142-3p could regulate the expression of *Bmal1* by directly targeting its 3’UTR.

### Mir-142 expression is under clock control

**Figure 3:**
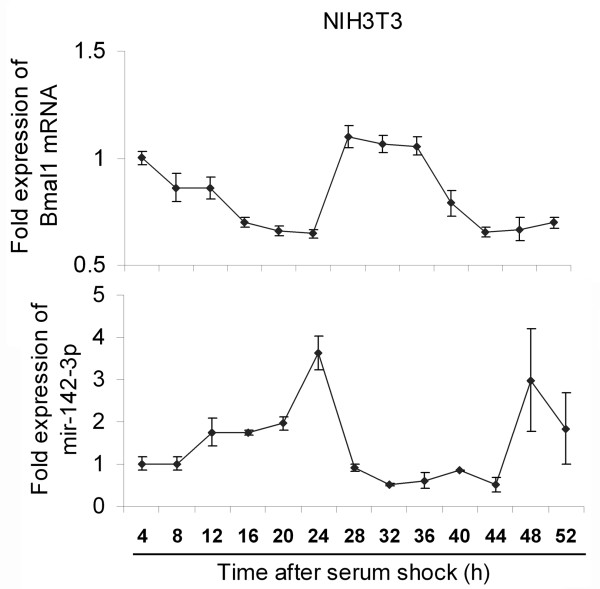
**The expression of mir-142-3p oscillates in serum shocked NIH3T3 cells. **The NIH3T3 cells were serum shocked and harvested every four hours for extraction of total RNA. And then the expression patterns of *Bmal1 *mRNA and mir-142-3p were determined by quantitative RT-PCR (mean ± SD, n = 3).

**Figure 4:**
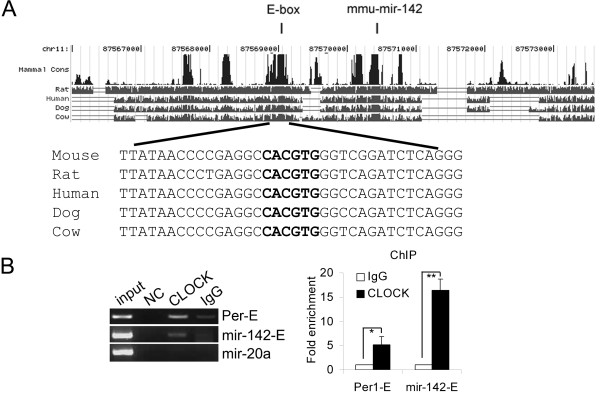
**The upstream regulatory sequence of mir-142 gene contains a CLOCK-binding E-box. **(**A**) A schematic representation of the conserved E-box element in the upstream regulatory sequence of the mouse mir-142 gene (obtained from UCSC Genome Browser Database). The arrow indicates the transcriptional orientation. Sequence alignments of mouse, rat, human, dog and cow (bottom) are shown. (**B**) Chromatin immunoprecipitation (ChIP) assays of NIH3T3 cells with ChIP-grade CLOCK antibody. The primers for amplifying the *Per1 *E-box or the upstream regulatory sequence of mir-20a gene were used as the positive and negative controls, respectively (left). The ChIP results were also quantified by quantitative real-time PCR (mean ± SD, n = 3). *P < 0.05 and **P < 0.01.

We have identified mir-142-3p as a potential regulator of *Bmal1*; therefore, it is of interest to explore the possibility that if this *Bmal1*-targeting miRNA is under circadian control. In a synchronized NIH3T3 cell model, we found that the level of mir-142-3p oscillated rhythmically, with the peak levels (24 h and 48 h) phase lagging the crests (4 h and 28 h) of *Bmal1* mRNA (Figure 3), suggesting the expression of mir-142-3p might be under circadian control. Interestingly, by analyzing the 5’ flank sequence of the mir-142 gene we found a conserved canonical E-box (CACGTG) (Figure 4A). The results of ChIP assays showed that CLOCK was able to bind to the E-box directly; the canonical E-box of *Per1* and the upstream sequence of the mir-20a gene were used as the positive and negative controls, respectively (Figure 4B).

**Figure 5:**
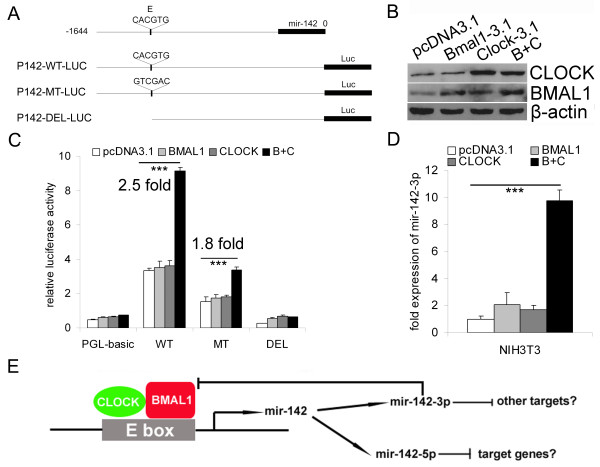
**CLOCK/BMAL1 heterodimers activate the expression of mir-142. **(**A**) The schematic representation for the luciferase reporter constructs. The upstream regulatory sequence of mir-142 together with pre-mir-142 was inserted upstream of the luciferase reporter gene. ‘E’ represents the conserved canonical E-box (CACGTG). ‘MT’ and ‘DEL’ denote the mutation (GTCGAC) and deletion of the E-box, respectively. (**B**) The over-expression of *Clock *and *Bmal1 *in NIH3T3 cells was confirmed by western blotting. Bmal1-3.1 (Bmal1-pcDNA3.1) and Clock-3.1 (Clock-pcDNA3.1) denote the *Bmal1 *and *Clock *expression plasmids. (**C**) A luciferase reporter assay was performed to determine whether CLOCK and BMAL1 heterodimers could enhance the transcription of the luciferase reporters (mean ± SD, n = 4). (**D**) After over-expressing *Clock *and *Bmal1 *in NIH3T3, the level of mir-142-3p was determined by quantitative RT-PCR (mean ± SD, n = 3). ***P < 0.001. (**E**) A simple model indicating a potential negative feedback loop involving both the activation of mir-142 transcription by CLOCK/BMAL1 heterodimers and the ensuing inhibition of *Bmal1 *expression by mir-142-3p.

For further study, a 1.6 kb upstream regulatory sequence of mir-142 gene containing the E-box together with pre-mir-142 was then used for a luciferase reporter assay (Figure 5A). Co-expression of *Clock* and *Bmal1* increased the luciferase activity of P142-LUC by approximately 2.5 fold. Mutating the conserved E-box significantly decreased the basal luciferase activity and reduced the induction (1.8 fold) of luciferase activity by co-expressing *Clock* and *Bmal1* (Figure 5B and C). Besides the conserved canonical E-box, there is an unconserved non-canonical E’-box in the upstream regulatory sequence of mir-142 gene which might contribute to the induction of luciferase activity of P142-MT-LUC by co-expression of *Clock* and *Bmal1* (Additional file 2). The results confirmed that the over-expression of *Clock* and *Bmal1* enhanced the transcription of mir-142. Moreover, the simultaneous ectopic expression of *Clock* and *Bmal1* (but not *Clock* or *Bmal1* alone) significantly induced the expression of mir-142-3p in NIH3T3 cells (Figure 5D).

Taken together, CLOCK/BMAL1 heterodimers can bind to the E-box in the upstream regulatory sequence and activate the expression of *mir-142*; one product of *mir-142*, mir-142-3p, can act as a negative regulator of *Bmal1* by targeting its 3’ UTR. A concise model describing this potential negative feedback loop is shown in Figure 5E.

## Discussion

In this study, we showed that the clock-controlled mir-142-3p can directly target its circadian activator, *Bmal1.* Although mir-142-3p has been reported to be a potential *Bmal1*-targeting miRNA based on a luciferase reporter assay [17], the present report is the first confirmation that mir-142-3p can directly regulate the expression of *Bmal1* both in mouse and human cells. By over-expression of mir-142-3p in NIH3T3 cells, we showed mir-142-3p can regulate the expression of *Bmal1*. However, a further study on the effects of knocking down mir-142-3p in appropriate murine cell models (with high level of mir-142-3p) will help to confirm the relationship between mir-142-3p and its target at the physiological conditions. Feedback loops formed by clock genes are thought to be the basis of the molecular clock. To our knowledge, this is the first report suggesting a potential negative feedback loop formed by miRNAs and clock genes. Considering the important role of *Bmal1* in circadian clock, we speculate that the molecular clock might be also fine tuned by the miRNA-mediated negative feedback.

Besides *Bmal1*, *Clock* was also predicted to be targeted by several miRNAs using three bioinformatic algorithms (Additional file 3A). The results of our luciferase reporter assay showed that six candidates (mir-20a, mir-106a, mir-106b, mir-148a, mir-182 and mir-301a) could be *Clock*-targeting miRNAs (Additional file 3B and C). Among them, mir-182 was reported to be a modulator of *CLOCK* in a recent study [20]. Although we did not perform additional experiments to confirm the interaction between the miRNAs and *Clock*, the regulation of *Clock* by miRNAs deserves further study. Indeed, a similar regulatory network consisting of miRNAs and *Clock* may exist, and a feedback loop involving mir-182/96 and *Clock* has been hypothesized [12].

In our study, we identified mir-142-3p as a regulator of *Bmal1* both in human and mouse cells. Even though we showed that *mir-142* is transcriptionally controlled by CLOCK/BMAL1 heterodimers only in a mouse fibroblast cell line (NIH3T3), the E-box in the upstream regulatory sequence of *mir-142* gene is perfectly conserved among mammals (Figure 4B). Thus, we hypothesize that this potential negative feedback mechanism could be conserved, at least between mice and human beings.

In our concise model, CLOCK/BMAL1 heterodimers enhance the transcription of *mir-142*, the product of which, in turn, inhibits the expression of *Bmal1*. In addition to *Bmal1*, mir-142-3p was reported to target several genes, including *ADCY9*, whose product controls cAMP levels [21]. Other genes were also predicted to be targets of mir-142-3p (Additional file 4), and two of them (*foxo1* and *Nr3c1*) are involved in the circadian clock [22,23]. The other product of mir-142, mir-142-5p, was also predicted to target numerous genes, the most interesting one of which is *Clock*, the other master regulator of the molecular clock (Additional file 4). Further studies are needed to confirm these prediction results and to uncover the regulatory role of the mir-142 gene in the circadian clock.

## Conclusions

Our study demonstrates that mir-142-3p can directly target the 3’UTR of *Bmal1*. In addition, the expression of mir-142-3p is controlled by CLOCK/BMAL1 heterodimers, suggesting a potential negative feedback loop consisting of the miRNAs and the core clock genes. These findings open new perspective for studying the molecular mechanism of circadian clock.

## Methods

### Plasmid DNA constructs

The mouse *Bmal1* and human *BMAL1* 3’UTRs were cloned from the cDNA of NIH3T3 and 293ET cells, respectively, by PCR. The PCR program was as follows: 95°C for 5 min; (95°C for 30 s; 55°C for 30 s and 72°C for 30 s) х30 and 72°C for 10 min. The mouse *Clock* 3’ UTR was cloned from cDNA obtained from the NIH3T3 cell line. For the mutagenesis experiment, the mir-142-3p binding site was replaced with a random sequence by bridge PCR. For the miRNA expression plasmids, the genomic sequences of the respective pre-miRNAs were amplified by PCR from mouse genomic DNA. The PCR program was as follows: 95°C for 5 min; (95°C for 30 s; 60°C for 30 s and 72°C for 30 s) х30 and 72°C for 10 min. The PCR products and respective vectors (pcDNA3.1) were digested directly with restriction enzymes. For cloning the *Bmal1/BMAL1* 3’ UTR, we used the pcDNA3.1-LUC or pcDNA3.1-EGFP vector, and the relevant enzymes were XhoI and XbaI. The pcDNA3.1-LUC and pcDNA3.1-EGFP vector are modified from pcDNA3.1 (+) (Invitrogen) as previously described [24,25]. For the miRNA expression plasmids, pcDNA3.1 (+) was chosen as the expression vector, and the relevant enzymes were BamHI and XhoI, with the exception of mir-20b (EcoRI and XhoI). All of the primers are listed in (Additional file 5).

### Cell culture and transfection

The 293ET cells were obtained from Dr. Chengyu Jiang (Peking Union Medical College). NIH3T3 was purchased from the American Type Culture Collection (ATCC). All of the cells were maintained in Dulbecco’s Modified Eagle Medium supplemented with 10% fetal bovine serum (FBS), 5 mM L-glutamine, and 100 U/ml penicillin and 100 mg/ml streptomycin. Transfections of plasmids, synthetic miRNA mimics and antagomirs were performed with a Lipofectamine 2000 Reagent (Invitrogen, Carlsbad, CA), according to the manufacturers protocol. The sequences of the synthetic mir-142-3p and mir-142-5p mimics are as follows. mir-142-3p: 5’-UGUAGUGUUUCCUACUUUAUGGA-3’; mir-142-5p: 5’-CAUAAAGUAGAAAGCACUACU-3’. The sequence of the synthetic mir-142-3p antagomirs is 5’-UCCAUAAAGUAGGAAACACUACA-3’. The synthetic miRNA mimics and antagomirs were purchased from Invitrogen.

### Luciferase and GFP reporter assay

The luciferase assays were performed using the Promega Dual-Luciferase Assay System. For testing the interactions between the *Bmal1*/*Clock* 3’ UTR and miRNAs, 200 ng of each of the *Bmal1*/*Clock* 3’ UTR-LUC constructs was co-transfected into 293ET cells with 50 ng of phRL-TK-TK (Renilla luciferase) for normalization and 1 μg of miRNA-pcDNA3.1 vector. For the transcriptional activity assay, NIH3T3 cells were transfected with 1.15 μg total DNA, including 100 ng of reporter vector (pGL3), 50 ng of phRL-TK, 500 ng of each expression plasmid (Bmal1-pcDNA3.1 or Clock-pcDNA3.1) and pcDNA3.1 to obtain a total amount of 1.15ug. After 48 hours, the cell lysates from all of the treatment groups were collected using Passive Lysis Buffer (Promega). The firefly luciferase activity was analyzed relative to the Renilla luciferase activity in the same sample using a Dual-Luciferase Reporter Assay System (Promega). The luminescence was measured using the GloMax Multi Detection System (Promega). For GFP reporter assay, 293ET cells were co-transfected with 100 ng of GFP reporter construct and 500 ng miRNA expression plasmid or control vector. 48 h later, cells were harvested for extraction of total RNA or protein. The GFP mRNA and protein level were determined by quantitative RT-PCR and Western blotting, respectively.

### Chromatin immunoprecipitation (ChIP) assay

The ChIP assays were performed as previously described [26]. Briefly, approximately 10^7^ NIH3T3 cells were cross-linked by incubation with 1% formaldehyde for 10 min at RT, and then the cross-linking reaction was stopped by adding glycine to a final concentration of 0.125 M with continued rocking for 5 min at room temperature. After washing, the cell pellet was suspended in nuclear lysis buffer, and each cross-linked sample was sonicated on ice and then incubated with anti-CLOCK (Calbiochem, Cat. No. 233170) or IgG. DNA was isolated from the immunoprecipitates and then subjected to PCR using the following primers: Mir-142-E, forward 5’-GTCGCTGGTTTCCTGTCAGT-3’ and reverse 5’-TGTCCGGAACCTCCACTTAG-3’; Mir-20a-E, forward 5’- TTGTGGTCCTGGCTCTCTCT-3’ and reverse 5’- ACTCACCCACTCAGGCAAAC-3’; and mper1-E1, forward 5'- GCTGACTGAGCGGTGTCTGA-3' and reverse 5'- GAGCGCCCTCCATCCGCTTG-3'.

### Quantitative real-time PCR

Total RNA was extracted from the NIH3T3, 293ET using the TRIZOL reagent (Invitrogen). TransScript First-Strand cDNA Synthesis SuperMix (TransGen Biotech) was used for reverse transcription. Stem-loop RT-PCR for the mature miRNAs was performed as previously described [27]. The miRNAs and the mRNAs of firefly luciferase, renilla luciferase and GFP were quantified with real-time PCR using SYBR® Premix Ex Taq™ (TaKaRa). The real-time PCR amplification was performed using 60 s of denaturation at 95°C and 45 cycles of 95°C for 5 s and 60°C for 34 s.

### Western blotting

Total cellular protein was separated by 10% sodium dodecyl sulfate polyacrylamide gel electrophoresis, and the proteins were transferred onto a nitrocellulose membrane. Then, the membrane was probed with rabbit anti-BMAL1 (Cat. sc-8550, Santa Cruz) or mouse anti-human β-actin antibodies (Cat. A5441, Sigma). After washing, the blots were treated with secondary antibodies conjugated to horseradish peroxidase and developed with ECL plus (Amersham).

### Serum shock procedures

The NIH3T3 cells were synchronized by serum shock as previously described [28]. Briefly, the NIH3T3 cells untreated or transfected with synthetic mir-142-3p mimics or control miRNA mimics were maintained in normal DMEM until the cells reached confluence. At time = 0, the medium was exchanged with serum-rich medium (supplemented with 50% FBS) and 2 h later the medium was replaced with serum-free DMEM. At the indicated times, the cells were harvest and kept in −70°C until the extraction of total RNA.

### Computational prediction

Three miRNA target prediction databases (TargetScan, PicTar and MicroCosm) were used to predict *Bmal1*/*Clock*-targeting miRNAs. The potential targets of mir-142-3p and -5p were predicted using the TargetScan and PicTar algorithms, and the targets predicted by both algorithms were listed in Additional file 4. The conserved E-box (CACGTG) in the upstream regulatory sequence of mir-142 was analyzed using DNAman, and the conservation track was obtained from the UCSC genome browser ( http://genome.ucsc.edu/).

### Statistical analysis

Data were expressed as mean ± SD. Statistical analyses were performed using a two-tailed unpaired t-test to compare data and a P-value less than 0.05 is considered statistically significant.

## Competing interests

The authors declare that they have no financial or non-financial competing interests.

## Authors' contributions

XCT and XZP conceived and designed the experiments; XCT, PZ and LZ performed the experiments; XCT, PZ, LZ and XZP analyzed the data; XCT, PZ, LZ, BY, HP and XZP contributed reagents/materials/analysis tools; XCT and XZP wrote the paper. All authors read and approved the final manuscript.

## Supplementary Material

Additional file 1 **mir-142 reduces the mRNA level of reporter genes. **(A) The over-expression of miRNAs in 293ET cells were determined by quantitative RT-PCR (mean ± SD, n = 3). (B) Firefly luciferase reporter plasmids containing *Bmal1/BMAL1 *3’ UTR and Renilla luciferase plasmid were co-transfected with mir-142-pcDNA3.1 expression plasmid or control vector into 293ET cells. 48 h later, cells were harvested for total RNA extraction. The Firefly and Renilla luciferase mRNA level were then measured by quantitative RT-PCR (mean ± SD, n = 3).*P < 0.05. Click here for file

Additional file 2 **There is an unconserved non-canonical E-box (E’-box) in the upstream regulatory sequence of mir-142 gene. **The E’-box is very close to the E-box and poorly conserved among mammals. Click here for file

Additional file 3 **Screening for miRNAs that can target Clock. **(A) Prediction results for miRNAs that might target clock by TargetScan, PicTar and MicroCosm. miRNAs that were predicted by at least two bioinformatic algorithms were listed in the table. (B) The schematic representation of the luciferase reporter construct containing *Clock *3’ UTR and the control vector. A 1.9 kb *Clock *3’ UTR containing all the binding sites of the candidate miRNAs was cloned downstream the luciferase cassette. (C) Luciferase reporter assay was performed to screen for potential Clock-targeting miRNAs. Data represent mean ± SD, n = 3. Two-tailed unpaired t test results are indicated by * for P < 0.05, relative to cells transfected with control vector. Click here for file

Additional file 4 **Predicting the targets of mir-142-3p and mir-142-5p. **The targets predicted by both TargetScan and PicTar were listed in the table. The targets involving in circadian clock are in gray letters. Click here for file

Additional file 5 **Primers for cloning *Bmal1***/***Clock *3’ UTR and pre-miRNAs and real-time PCR primers for detecting miRNAs and genes were listed in this table.** Click here for file
