# Supplementary figures and images for: Clock-controlled mir-142-3p can target its activator, Bmal1
Source: BMC Mol Biol. 2012 Sep 7;13:27. doi: 10.1186/1471-2199-13-27 (PMC3482555; doi:10.1186/1471-2199-13-27)

**A**


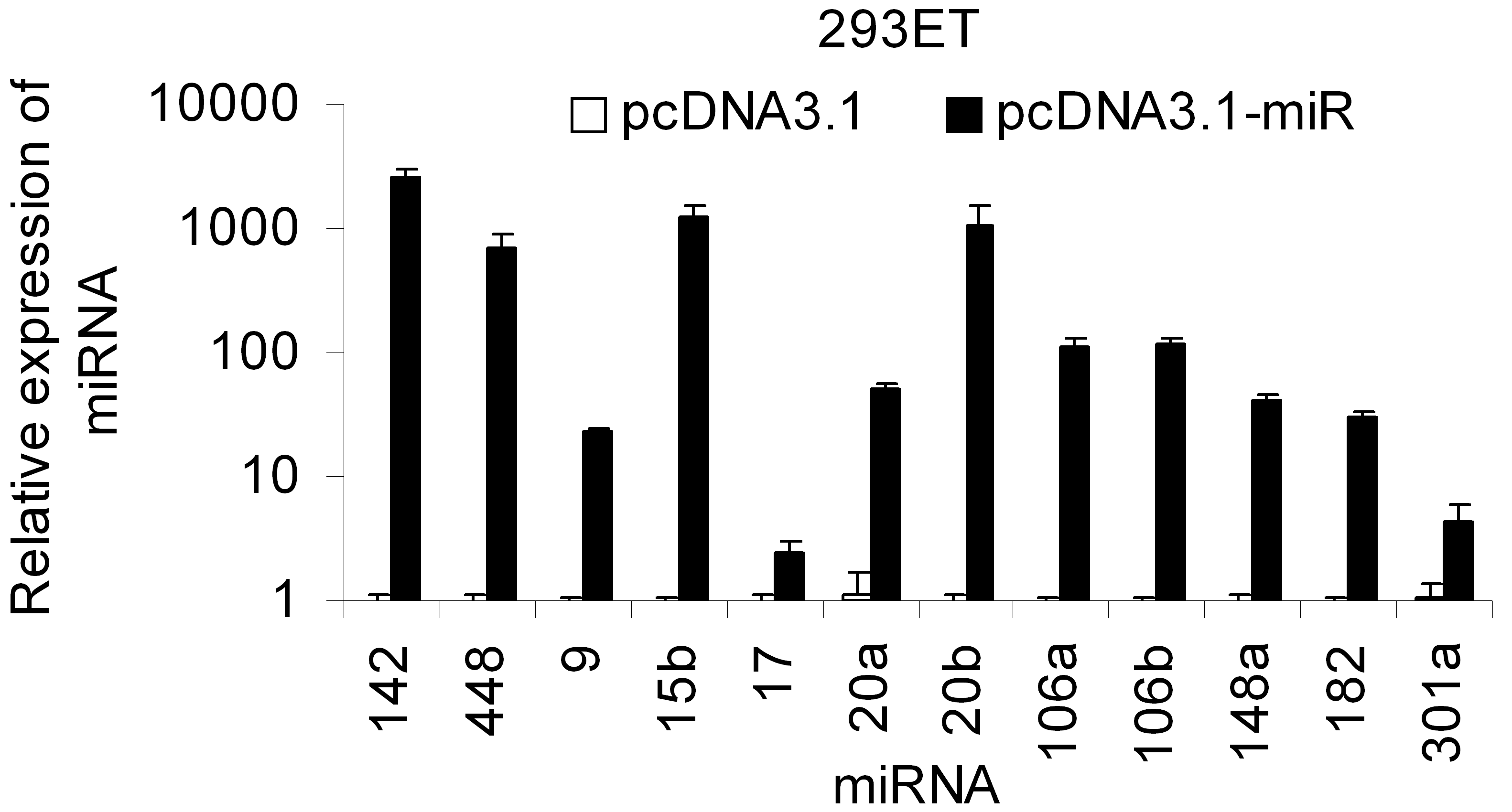


**B**


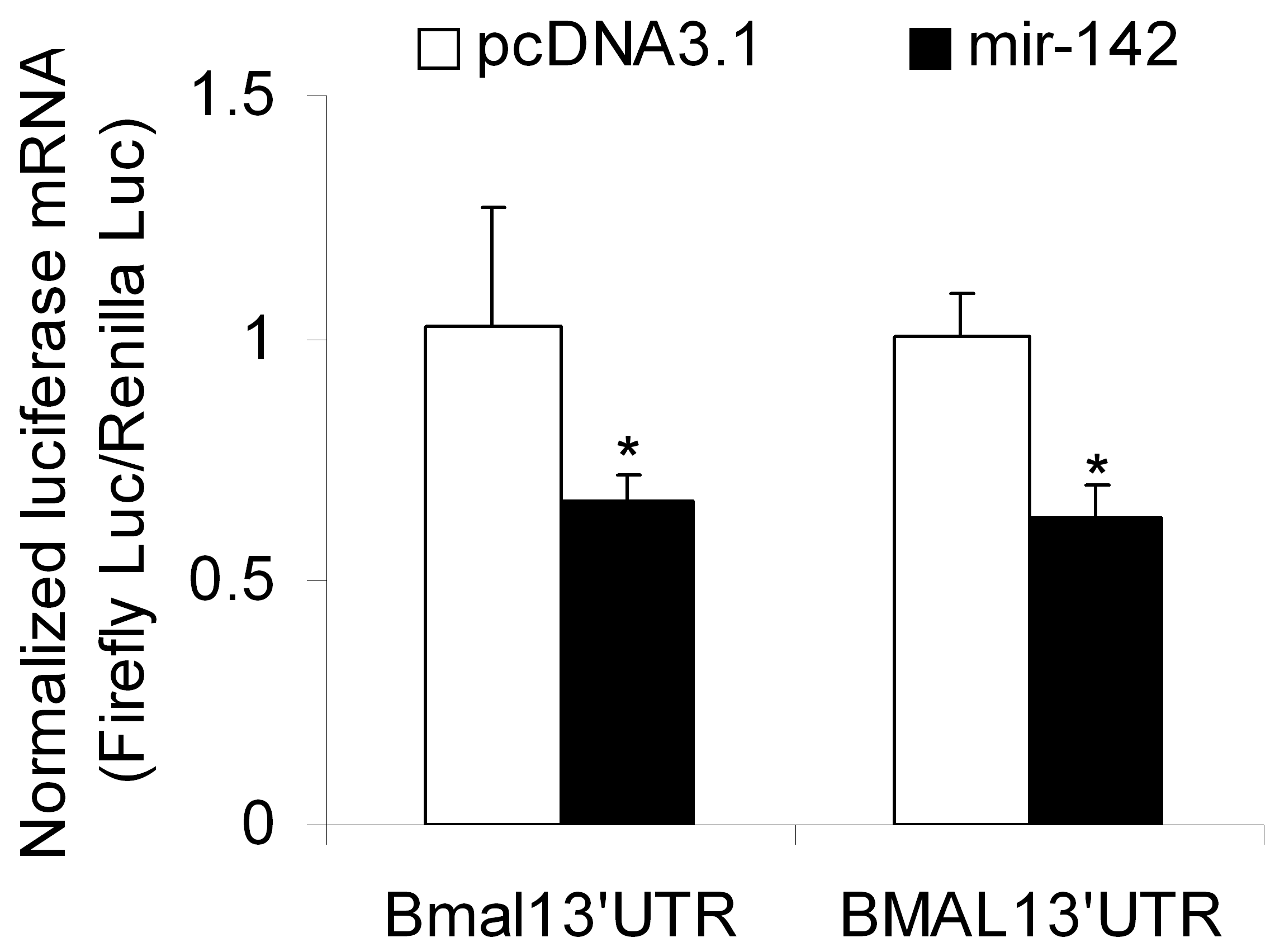

Supplement: Additional file 1 — mir-142 reduces the mRNA level of reporter genes. (A) The over-expression of miRNAs in 293ET cells were determined by quantitative RT-PCR (mean ± SD, n = 3). (B) Firefly luciferase reporter plasmids containing Bmal1/BMAL1 3’ UTR and Renilla luciferase plasmid were co-transfected with mir-142-pcDNA3.1 expression plasmid or control vector into 293ET cells. 48 h later, cells were harvested for total RNA extraction. The Firefly and Renilla luciferase mRNA level were then measured by quantitative RT-PCR (mean ± SD, n = 3).*P < 0.05. [file 1471-2199-13-27-S1.doc]

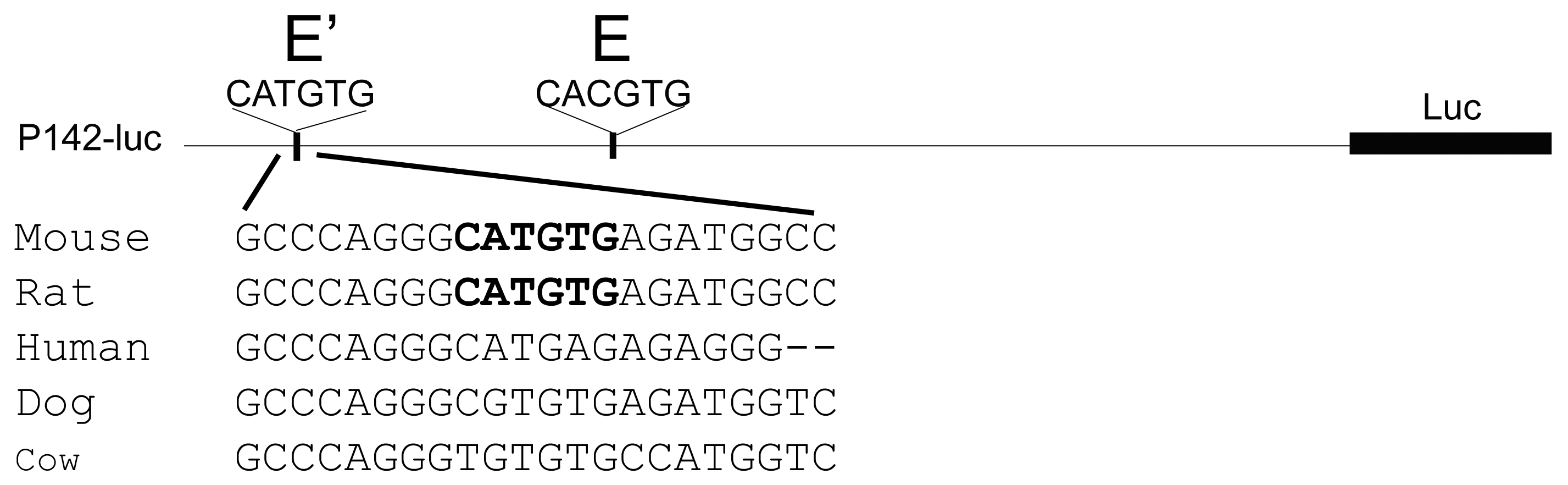

Supplement: Additional file 2 — There is an unconserved non-canonical E-box (E’-box) in the upstream regulatory sequence of mir-142 gene. The E’-box is very close to the E-box and poorly conserved among mammals. [file 1471-2199-13-27-S2.doc]

A


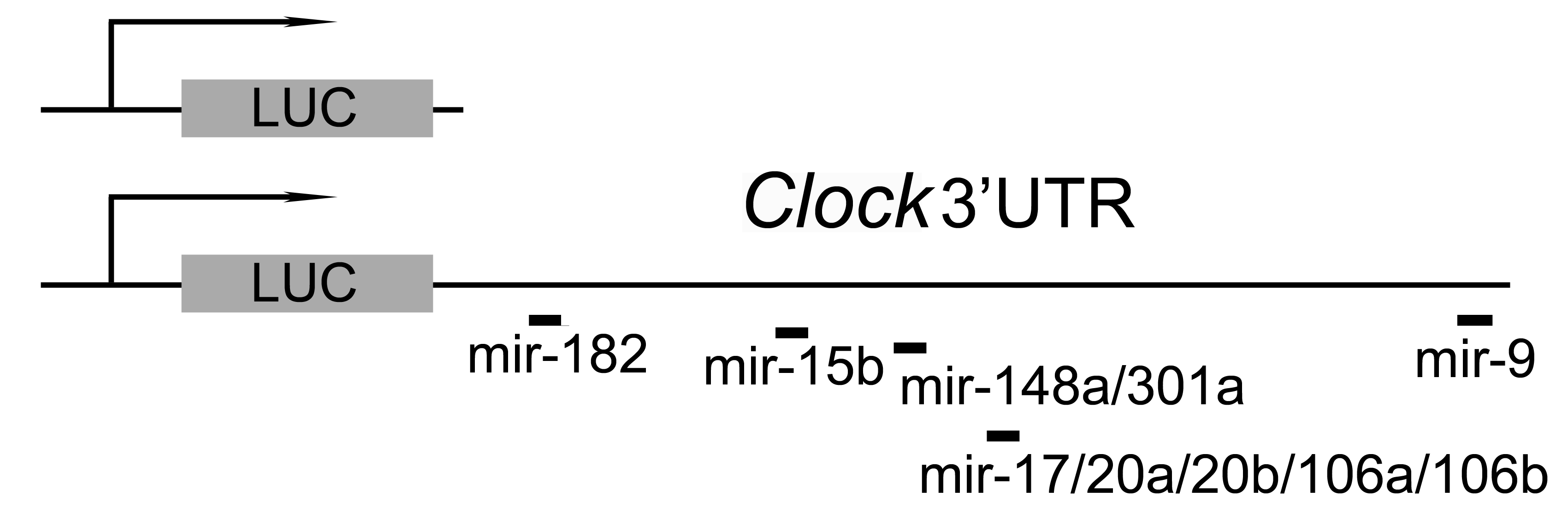
B

C


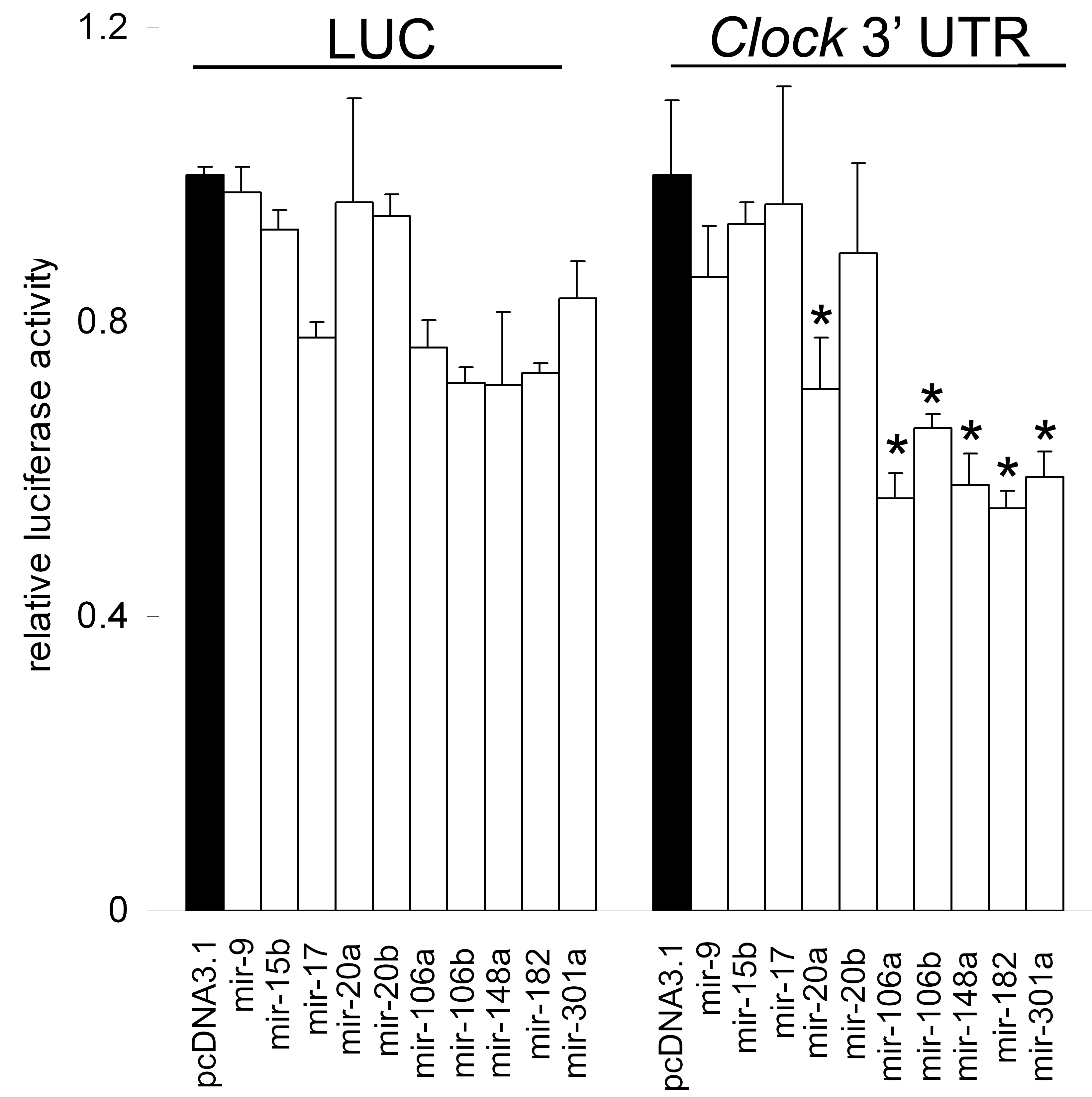

Supplement: Additional file 3 — Screening for miRNAs that can target Clock. (A) Prediction results for miRNAs that might target clock by TargetScan, PicTar and MicroCosm. miRNAs that were predicted by at least two bioinformatic algorithms were listed in the table. (B) The schematic representation of the luciferase reporter construct containing Clock 3’ UTR and the control vector. A 1.9 kb Clock 3’ UTR containing all the binding sites of the candidate miRNAs was cloned downstream the luciferase cassette. (C) Luciferase reporter assay was performed to screen for potential Clock-targeting miRNAs. Data represent mean ± SD, n = 3. Two-tailed unpaired t test results are indicated by * for P < 0.05, relative to cells transfected with control vector. [file 1471-2199-13-27-S3.doc]

Table S1 Prediction for the targets of mir-142-3p and -5p by TargetScan and PicTar.


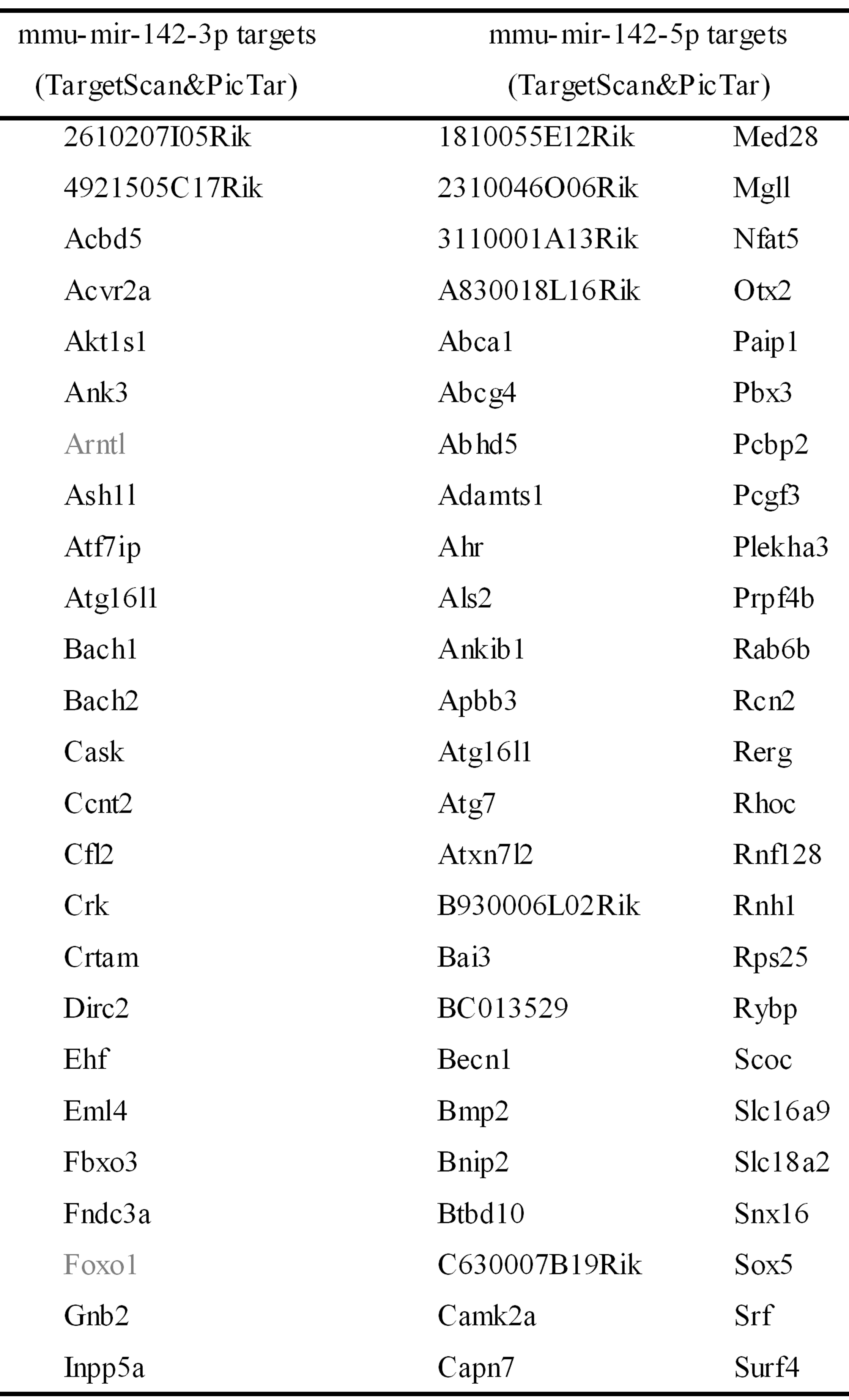


Table S1-continued


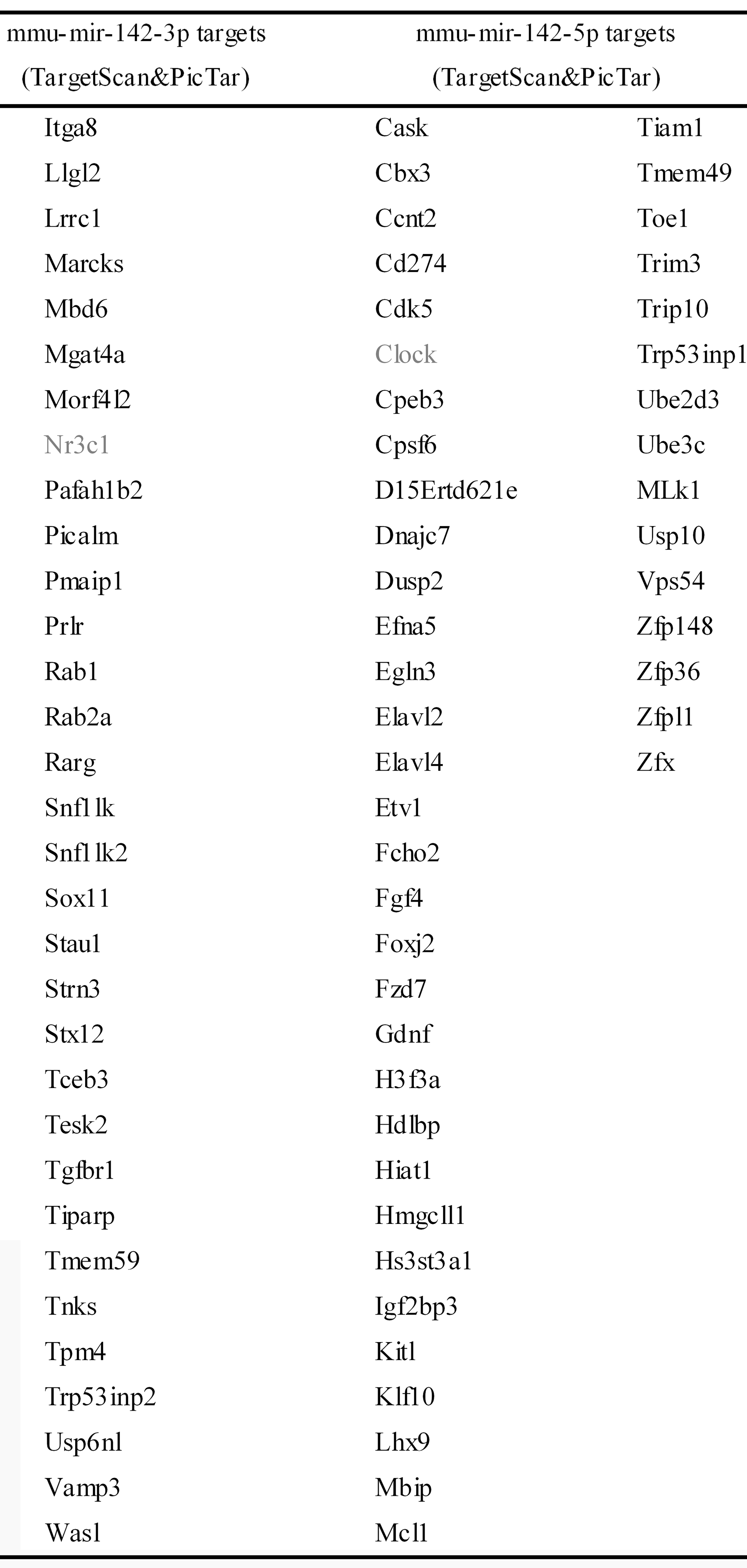

Supplement: Additional file 4 — Predicting the targets of mir-142-3p and mir-142-5p. The targets predicted by both TargetScan and PicTar were listed in the table. The targets involving in circadian clock are in gray letters. [file 1471-2199-13-27-S4.doc]
