# Supplementary material for: Clock-controlled mir-142-3p can target its activator, Bmal1
Source: BMC Mol Biol. 2012 Sep 7;13:27. doi: 10.1186/1471-2199-13-27 (PMC3482555; doi:10.1186/1471-2199-13-27)
Supplement: Additional file 5 — Primers for cloning Bmal1/Clock 3’ UTR and pre-miRNAs and real-time PCR primers for detecting miRNAs and genes were listed in this table. [file 1471-2199-13-27-S5.doc]

Table S2 Primers used in the study.

A. primers used for cloning the 3’UTR of *Bmal1* and *Clock*

| 3'UTR | Primers for amplifying the 3'UTR (5'-3') |
| --- | --- |
| Bmal13'UTR(mouse) | |
| forward | CCGCTCGAGGTTGACTTTAGTGACTTGCCA |
| reverse | TGCTCTAGAAGAACAAGGGAAACATTTATTAAAAATA |
| Bmal13'UTR(human) | |
| forward | CCGCTCGAGGCTGTAAACACTACATGTTGCTTTG |
| reverse | GCTCTAGAGAACAAGGGAAACATTTATTAAAAATATTTAACTG |
| Clock3'UTR(mouse) | |
| forward | CCGCTCGAGGCACACACACTTCCTCTCTGAC |
| reverse | GAAGCAGTCCAGAGCAAGGTAC |
| Bmal13'UTR(mouse)-mutaion1 | |
| forward | CCGCTCGAGGCGCGACGCTTTGCTTTGGCAACAGCTG |
| reverse | TGCTCTAGAAGAACAAGGGAAACATTTATTAAAAATA |
| Bmal13'UTR(mouse)-mutation2  -half1-reverse | TCCACATGGGGGACTTCTTGCGTCGCGAAAAACACCATACATCTGAAATGAC |
| Bmal13'UTR(mouse)-mutation2  -half2-forward | GTCATTTCAGATGTATGGTGTTTTTCGCGACGCAAGAAGTCCCCCATGTGGA |

B. primers for cloning the pre-miRNAs.

| miRNAs |  | primers for amplifying the pre-miRNAs (5'-3') |
| --- | --- | --- |
| mmu-miR-142-3p | forward | CGGGATCCCCGTGGACAGACAGACAGTG |
|  | reverse | CCGCTCGAGGTATCAGGGGTCAGGAAGCAC |
| mmu-miR-448 | forward | CGGGATCCAGTACTCAATGCCATTCCATTCAG |
|  | reverse | CCGCTCGAGGGCTCTTCAGTCAAGCAGAATG |
| mmu-miR-15b | forward | CGGGATCCAGAAGCCATGGCATTGACTTAG |
|  | reverse | CCGCTCGAGTAGAGTGGAACAAGCATGTCAG |
| mmu-miR-20a | forward | CGGGATCCCTGGATGCAAACCTGCAAAAC |
|  | reverse | CCGCTCGAGTCAGTCCACCAGGGTAAGAAG |
| mmu-miR-20b | forward | GGAATTCGAACGGCACAAGTTTCAAAGG |
|  | reverse | CCGCTCGAGCCCACAATCAGTTTTGCATGG |
| mmu-miR-106a | forward | CGGGATCCGTGTGTGTGTGTGTTTATCCAG |
|  | reverse | CCGCTCGAGACACATTACAAGGAGCAGCTC |
| mmu-miR-106b | forward | CGGGATCC TCTCCGACTTTCCACTGCTC |
|  | reverse | CCGCTCGAGGAGGGGAGGACAGAATGGAG |
| mmu-miR-148a | forward | CGGGATCCGCAGGACGAAACTTCCAGAG |
|  | reverse | CCGCTCGAGTCAGTCCACCAGGGTAAGAAG |
| mmu-miR-182 | forward | CGGGATCCCCGAAGGACCATAGTCTGG |
|  | reverse | CCGCTCGAGCCAAGTCCTTTTCACCGAG |
| mmu-miR-301a | forward | CGGGATCCTCTTTGCACACTGAGCCTTG |
|  | reverse | CCGCTCGAGGTAATGAATTTTCAGTGCCATACAG |

C. primers for real-time PCR

| **miRNAs** |  | real-time PCR primers (5'-3') |
| --- | --- | --- |
|  | reverse (universal) | GTGCAGGGTCCGAGGT |
| mmu-miR-142-3p | RT | GTCGTATCCAGTGCAGGGTCCGAGGTATTCGCACTGGATACGACTCCATA |
|  | forward | CGCGTGTAGTGTTTCCTACTTTATG |
| mmu-miR-448 | RT | GTCGTATCCAGTGCAGGGTCCGAGGTATTCGCACTGGATACGACATGGGA |
|  | forward | CGCGTTGCATATGTAGGATGTC |
| mmu-miR-15b | RT | GTCGTATCCAGTGCAGGGTCCGAGGTATTCGCACTGGATACGACTGTAAAC |
|  | forward | CCGGGTAGCAGCACATCATG |
| mmu-miR-20a | RT | GTCGTATCCAGTGCAGGGTCCGAGGTATTCGCACTGGATACGACCTACCT |
|  | forward | GGGGGTAAAGTGCTTATAGTGC |
| mmu-miR-20b | RT | GTCGTATCCAGTGCAGGGTCCGAGGTATTCGCACTGGATACGACCTACCT |
|  | forward | GCCCCCAAAGTUGCTCATAGT |
| mmu-miR-106a | RT | GTCGTATCCAGTGCAGGGTCCGAGGTATTCGCACTGGATACGACCTACCT |
|  | forward | GGGGCCAAAGTGCTAACAGT |
| mmu-miR-106b | RT | GTCGTATCCAGTGCAGGGTCCGAGGTATTCGCACTGGATACGACATCTGC |
|  | forward | CGCGTAAAGTGCTGACAGTG |
| mmu-miR-148a | RT | GTCGTATCCAGTGCAGGGTCCGAGGTATTCGCACTGGATACGACACAAAG |
|  | forward | CGCGTCAGTGCACTACAGAAC |
| mmu-miR-182 | RT | GTCGTATCCAGTGCAGGGTCCGAGGTATTCGCACTGGATACGACCGGTGT |
|  | forward | CGCGGTTTGGCAATGGTAGA |
| mmu-miR-301a | RT | GTCGTATCCAGTGCAGGGTCCGAGGTATTCGCACTGGATACGACGCTTTG |
|  | forward | CGCGCAGTGCAATAGTATTGTC |
| mmu-miR-17 | RT | GTCGTATCCAGTGCAGGGTCCGAGGTATTCGCACTGGATACGACCTACCT |
|  | forward | CCGCCCAAAGTGCTTACAGT |
| mmu-mir-9 | RT | GTCGTATCCAGTGCAGGGTCCGAGGTATTCGCACTGGATACGACTCATAC |
|  | forward | CGGCCGTCTTTGGTTATCTAGC |
| U6 | RT | GTCGTATCCAGTGCAGGGTCCGAGGTATTCGCACTGGATACGACAAAATATG |
|  | forward | GCGCGTCGTGAAGCGTTC |
|  |  |  |
| **Genes** |  |  |
| Bmal1 | forward | TGGCCGCTGTAGACACTACATT |
|  | reverse | CTCTATCCAGTAAGCTTCACAGACTGTAA |
| Per1 | forward | AGAAGAAAACAGCACCAGCT |
|  | reverse | TCTTGAGTTATAAGAACCCCAACATG |
| BMAL1 | forward | GCCCATTGAACATCACGAGTAC |
| Fluc | forward | CTGATTTTTCTTGCGTCGAGTTT |
|  | reverse | GCGCGGAGGAGTTGTGTTT |
| Rluc | forward | ACATGGTAACGCGGCCTCTT |
|  | reverse | TGCCCATACCAATAAGGTCTGGTA |
| gfp | forward | AAGCTGACCCTGAAGTTCATCTG |
|  | reverse | GTGCGCTCCTGGACGTAG |
